# *SacB-SacR* Gene Cassette As the Negative Selection Marker to Suppress *Agrobacterium* Overgrowth in *Agrobacterium*-Mediated Plant Transformation

**DOI:** 10.3389/fmolb.2016.00070

**Published:** 2016-10-26

**Authors:** Yiming Liu, Jiamin Miao, Sy Traore, Danyu Kong, Yi Liu, Xunzhong Zhang, Zachary L. Nimchuk, Zongrang Liu, Bingyu Zhao

**Affiliations:** ^1^Department of Crop and Soil Environmental Science, Virginia TechBlacksburg, VA, USA; ^2^Chinese Academy of Tropical Agricultural Sciences/Key Laboratory of Crop Gene Resources and Germplasm Enhancement in Southern China, Tropical Crops Genetic Resources Institute, Ministry of AgricultureDanzhou, China; ^3^Department of Horticulture, Virginia TechBlacksburg, VA, USA; ^4^Department of Biology, University of North Carolina at Chapel HillChapel Hill, NC, USA; ^5^USDA-ARS-Appalachian Fruit Research StationKearneysville, WV, USA

**Keywords:** plant transformation, *Agrobacterium* overgrowth, CRISPR-*Cas9*, *Nicotiana benthamiana*, *SacB-SacR* gene cassette

## Abstract

*Agrobacterium* overgrowth is a common problem in *Agrobacterium*-mediated plant transformation. To suppress the *Agrobacterium* overgrowth, various antibiotics have been used during plant tissue culture steps. The antibiotics are expensive and may adversely affect plant cell differentiation and reduce plant transformation efficiency. The SacB-SacR proteins are toxic to most *Agrobacterium tumefaciens* strains when they are grown on culture medium supplemented with sucrose. Therefore, *SacB-SacR* genes can be used as negative selection markers to suppress the overgrowth of *A. tumefaciens* in the plant tissue culture process. We generated a mutant *A. tumefaciens* strain GV2260 (*recA-SacB/R*) that has the *SacB-SacR* cassette inserted into the bacterial genome at the *recA* gene locus. The mutant *Agrobacterium* strain is sensitive to sucrose but maintains its ability to transform plant cells in both transient and stable transformation assays. We demonstrated that the mutant strain GV2260 (*recA-SacB/R*) can be inhibited by sucrose that reduces the overgrowth of *Agrobacterium* and therefore improves the plant transformation efficiency. We employed GV2260 (*recA-SacB/R*) to generate stable transgenic *N. benthamiana* plants expressing a CRISPR-*Cas9* for knocking out a WRKY transcription factor.

## Introduction

*Agrobacterium*-mediated genetic transformation is one of the most popular techniques used for the generation of transgenic plants (Gelvin, 2000; Tzfira and Citovsky, 2006). Efficient *Agrobacterium*-mediated transformation protocols have been developed for various plant species (Hiei et al., 1994; Ishida et al., 1996; Cheng et al., 1997; Tingay et al., 1997; Zhao et al., 2000). In general, the *Agrobacterium*-mediated transformation involves the generation of a sterile explant that can be co-cultured with *Agrobacterium.* Subsequently, the infected *Agrobacterium* cells are eliminated or suppressed by using various antibiotics, and the transgenic plant cells are selected by using antibiotics or other chemicals (Jones et al., 2005; Tsuda et al., 2012). During the *Agrobacterium*-mediated transformation process, one major problem is the overgrowth of *Agrobacterium* that could significantly reduce the plant transformation efficiency. To eliminate or inhibit the *Agrobacterium* overgrowth, different antibiotics such as carbenicillin, Timentin™, Augement, Clavamox, and Cefotaxime are used during the plant tissue culture selection steps (Bhau and Wakhlu, 2001; Tereso et al., 2006; Zang et al., 2009; Li and Qu, 2011; Ren et al., 2012). For example, carbenicillin is a semi-synthetic penicillin antibiotic that interferes with cell wall mucopeptide biosynthesis of gram-negative bacteria (Silva and Fukai, 2001). Under selection pressure, *Agrobacterium* cells could gain mutations that are resistant to the carbenicillin, which results in the overgrowth of *Agrobacterium*. Most antibiotics are generally expensive and may negatively affect plant cell differentiation (Ellis et al., 1989; Yu et al., 2001; Li and Qu, 2011). Therefore, a more reliable and cost-effective method to inhibit the overgrowth of *Agrobacterium tumefaciens* is highly desirable.

The *SacB-SacR* genes were originally isolated from *Bacillus subtilis* and encode levansucrase, an enzyme involved in both the hydrolysis of sucrose and the biosynthesis of levan (Chambert and Petitglatron, 1989; Quandt and Hynes, 1993; Traore and Zhao, 2011). Levan cannot be metabolized by most gram-negative bacteria including *A. tumefaciens* and is therefore toxic to this group of organisms (Gay et al., 1985; Schweizer, 1992). The *SacB-SacR* gene cassette, driven by its native promoter, has been used as a negative selectable marker for many gram-negative bacteria, and works by preventing the transformed bacterial cells from growing on culture medium supplemented with sucrose (Ried and Collmer, 1987). Our previous work demonstrated that the *SacB-SacR* genes can be used as negative selection markers to inhibit the growth of *Agrobacterium* strain GV2260 on Luria Broth agar medium supplemented with 5% sucrose (Traore and Zhao, 2011). Sucrose has been frequently used as the carbon source in synthetic plant tissue culture medium (Yaseen et al., 2013), although other sugars such as maltose, fructose, and sorbitol have also been used. It is interesting to test if growth of an *Agrobacterium* strain carrying the *SacB-SacR* gene cassette can be inhibited on plant tissue medium supplemented with sucrose.

The *recA* gene was originally identified as a conserved gene involved in homologous DNA recombination in various bacterial species (Clark and Margulies, 1965; Brendel et al., 1997; Song et al., 2003). *RecA*-dependent recombination was initially identified through analysis of conjugational recombination (Clark and Margulies, 1965; Bi and Liu, 1994), where *recA* can promote homologous pairing of DNA molecules and catalyzes the strand exchange reaction leading to the formation of hetero-duplex DNA *in vitro* (West, 1992; Bi and Liu, 1994). Deletion of the *recA* gene in the bacterial genome can reduce the rate of homologous recombination, and therefore increase plasmid DNA stability. Deletion of the *recA* gene in *E. coli* has no obvious deleterious effect on bacterial growth (Kurnit, 1989; Lovett et al., 1993). Several *Agrobacterium* strains with deletion of the *recA* gene were also developed (Farrand et al., 1989).

In this study, we attempted to integrate the *SacB-SacR* gene cassette at the *recA* gene locus in the genome of *A. tumefaciens* strain GV2260. The derived mutant strain GV2260 (*recA-SacB/R*) was used to transform *Nicotiana benthamiana* (*N. benthamiana*) plant cells in both transient assays and stable transformation. We demonstrated that the mutant strain GV2260 (*recA-SacB/R*) maintains its capacity of transforming plant cells, and its growth can be efficiently inhibited by regular tobacco tissue culture medium supplemented with 3% sucrose. Stable transgenic plants carrying a CRISPR-*Cas9* construct for knocking out a WRKY transcription factor were successfully recovered after transformation with the mutant *Agrobacterium* strain. Therefore, the mutant *A. tumefaciens* strain should have great value for large scale, high-throughput plant transformation applications in the future.

## Materials and methods

### Bacteria strains

*Escherichia coli* (*E. coli*) DH5α [F^−^endA glnV44 thi-1 recA1 relA1 gyrA96 deoR nupG Φ80dlacZΔM15Δ(lacZYA-argF)U169, hsdR17(${\text{r}}_{\text{K}}^{-}$${\text{m}}_{\text{K}}^{+}$), λ^−^], *A. tumefaciens* (GV2260) [C58 background, rifampicin-resistant with the Ti plasmid (pTiB6s3)], and *Escherichia coli* (*E. coli*) helper P600 (Traore and Zhao, 2011).

### Plant materials

*N. benthamiana* (PI 555478) plants were propagated in a growth chamber programmed for 16 h light (140 μmol m^−2^s^−1^ cool white fluorescent irradiance) at 28°C and 8 h dark at 24°C *Agrobacterium*-mediated transient assays were conducted on three- to 4-week-old plants.

### Cloning of the *Agrobacterium recA* gene fragment

A 780 bp DNA fragment with deletion of the N and C-terminus of the *recA* gene was amplified from the genomic DNA of GV2260 using primers: *recA* For, 5′-caccatcgatcatgaagctcggt-3′ and *recA* Rev, 5′-gcgccggacttctcgacgat-3′. The PCR reaction as performed using the iProof™ high fidelity Taq DNA polymerase (Bio-Rad, Hercules, CA). The PCR program consisted of 1 cycle at 98°C (2 min), followed by 30 cycles at 98°C (30 s), 55°C (45 s), and 72°C (1 min), and finished with a 1 cycle extension at 72°C (7 min). The PCR product was separated on a 0.8% agarose gel, stained with 0.01% ethidium bromide solution, and visualized using the Gel-Document Image System™ under UV light (Bio-Rad).

The PCR product was purified using the AccuPrep™ Gel Purification Kit (Bioneer, Alameda, CA) and cloned into the TopoEntr/D™ vector (Invitrogen, Carlsbad, CA) following the instructions of the user manual. The derived plasmid vector was designated as TopoEntr-*recA* and has been sequenced at the core facility of the Virginia Bioinformatics Institute (Blacksburg, VA).

### Development of an integrational construct carrying *rec*A fragment and the *SacB-SacR* gene cassette

The suicide vector pLVC18L (Zhao et al., 2011) was modified by insertion of the *SacB/R* and the *ccd*B gene cassettes. The *Npt*2 promoter-*SacB/R* fragment was amplified through overlap PCR from pEG101-*SacB/R* and pDSK519-GFP (Matthysse et al., 1996; Traore and Zhao, 2011) using primers: 1846pLvc18 XbaNpt2 Infusion For1, 5′-TGC CATTGCTGCAGGTCGACTCTAGAGATATCACATGGCGATAGCTAGACT G-3′; 1777Npt2Pro_*SacB/R* Rv Rev1, 5′-GTG ATGGGTTAAAAAGGATCGATCCGCGCCATCAGATCC TTG-3′; 1778Npt2Pro_*SacB/R* Rv For2, 5′-CAA GGATCTGATGGCGCGGATCGATCCTTTTTAACCCAT CAC-3′; 1847pLvc18 XbaSacB Infusion Rev2, 5′-CTC GGTACCCGGGGATCCTCTAGAGATATCTTATTTGTTAACTGTTAATTG TCCT-3′.

The PCR product was cloned into the *Xho*I site of pLVC18L using a Gibson cloning kit (New England BioLabs Inc., Ipswich, MA). The derived construct was designated as pLVC18L-Npt2-*SacB/R*. A *ccd*B gene cassette (frame B) (Invitrogen) was further cloned into the *Sma*I site of pLVC18L-Npt2-*SacB/R* to generate pLVC18L-Npt2-*SacB/R*-DesB. The *recA* gene fragment from TopoEntr-*recA* was subcloned into pLVC18L-Npt2-*SacB/R*-DesB using a Gateway®; LR cloning kit (Invitrogen) following the instructions of the user manual. The derived plasmid construct was named pLVC18L-Npt2-*SacB/R*-*recA*, and has been confirmed by sequencing at the core facility of the Virginia Bioinformatics Institute (Blacksburg, VA).

### Integration of the pLVC18L-Npt2-*SacB/R*-*recA* construct into the genome of *Agrobacterium tumefaciens* strain GV2260

The suicide vector pLVC18L-Npt2-*SacB/R*-*recA* was integrated to the genome of *A. tumefaciens* strain GV2260 by tri-parental conjugation and was selected on LB medium supplemented with tetracycline (10 μg/mL) as previously described (Traore and Zhao, 2011). A mutant GV2260 strain carrying the *Npt*2-*SacB/R* cassette was confirmed by PCR amplification of the tetracycline resistance gene and *SacB/R* gene using primers: tetracycline For, 5′-atgaaatctaacaatgcg ctcat-3′; tetracycline Rev, 5′-tacgagttgcatgataaagaa gaca-3′, and *SacB-SacR* For, 5′-cagcatatcatggcgtgt aatatg-3′; *SacB-SacR* Rev, 5′-ctcggtacccggggatcctctagagat atcttatttgttaactgttaattgtcct-3′. The derived mutant strain was designated as GV2260-*SacB/R*.

### Development of the plasmid vector pEarleygate101-YFP-HA

The YFP gene open reading frame plus the HA epitope tag was amplified from vector pEarleygate101 (Earley et al., 2006) with primers 2702pEG101-yfpHA For, 5′-ATTTGGAGAGGACACG**ctcgag**AtgAGCAAGGGCGAGGAGCTGTTC ACCG-3′; 2703pEG101-yfpHA Rev, 5′-TCGACTGCAGAATTCGAAGCTTGAG**ctcgag**ATCTGAG-3′. The PCR product was gel purified and cloned into pEarleygate101 that had been digested with *Xho*I. The derived construct was designated as pEarleygate101-YFP-HA.

### Development of a CRISPR-*Cas*9 construct pgRNA-*NbWRKY70* for knocking out tobacco transcription factor WRKY70

A putative tobacco WRKY transcription factor NbWRKY70 was identified from GenBank (accession number AF421157). To knock out NbWRKY70, we identified a guiding RNA (GCAATCGACGGGTTAATTCG**CGG**) targeting the *NbWRKY70* gene. An *Arabidopsis* U6 promoter, NbWRKY70 guiding RNA, and the PAM terminator were amplified through overlap-PCR using primers 2284AtU6gRNA common For1, CAGCAACTCATTACAACTTGTTTaagctttcgttgaacaacgga; 2285AtU6gRNA common Rev1, CGACTCTAGACACGGGGTGGTTTaaaaaaagcaccgactcggtgcc; 2846NbWRKY70 gRNA For, GCAATCGACGGGTTAATTCGgttttagagctagaaatag; 2847NbWRKY70 gRNA Rev, CGAATTAACCCGTCGATTGCaatcactacttcgactcta.

The PCR product was gel purified and cloned into the *Pme*I site of pM3UT-Cas9 using a Gibson cloning kit (New England BioLabs), where it contains a *Cas9* gene driven by the *Arabidopsis* Ubiquitin 10 promoter. The *Cas9* gene was originally codon optimized and synthesized based on the Arabidopsis genes (Zachary Nimchuk, unpublished data). The derived construct was designated as pgRNA-*NbWRKY70*.

### Conjugation of pEarleygate101-YFP-HA and pgRNA-*NbWRKY70* into *Agrobacterium tumefaciens* strain GVV2260 and GV2260-*SacB/R*

The plasmid vectors pEarleygate101-YFP-HA and pgRNA-*NbWRKY7*0 were conjugated into *A. tumefaciens* strain GVV2260 and GV2260-*SacB/R* using tri-parental conjugation, selected on LB medium supplemented with rifampin 100 μg/mL, and Kanamycin 50 μg/mL or Spectinomycin 50 μg/mL as previously described (Traore and Zhao, 2011).

### *Agrobacterium*-mediated transient assays in *N. benthamiana* plants

*Agrobacterium*-mediated transient assays in *N. benthamiana* plants were performed as described previously (Wydro et al., 2006). In brief, the *Agrobacterium* strains were streaked on Yeast Extract Tryptone (YT) media supplemented with rifampicin 100 μg/mL, tetracycline 10 μg/mL, and kanamycin 50 μg/mL and incubated at 28°C for 2 days. Bacterial cells were harvested and re-suspended in induction buffer composed of 10 mM MgCl_2_, 10 mM MES (pH 5.6), and 100 μM acetosyringone and incubated for 3 h at room temperature. The bacterial inoculums were adjusted to OD_600_ nm = 0.6 and infiltrated into the stomata of the fully expanded *N. benthamiana* leaves using a 1-mL blunt-end syringe without a needle. The inoculated plants were incubated at room temperature under continuous light for 20–48 h before the detection of expressed proteins. The fluorescent signal of YFP-HA fusion protein was monitored 24 h after inoculation by fluorescent microscopy (Zeiss Axio Observer.A1, Carl Zeiss MicroImaging, Inc., Thornwood, NY).

### Generation of transgenic tobacco plants using either wild type or mutant *Agrobacterium* strains

*Agrobacterium* strain GV2260 or GV2260-*SacB/R* carrying plasmid pgRNA-*NbWRKY70* were used for tobacco transformation following a previously described protocol (Horsch et al., 1989). In brief, the fully expanded leaf from a 4-week-old *N. benthamiana* plant was collected and sterilized in 10% bleach for 20 min. The leaf was cut into 1 cm^2^ leaf disks that were infected with *Agrobacterium* culture diluted to OD_600_ = 0.1. The infected leaf disks were co-cultured on MS medium supplemented with 6-BA (1 mg/L) and NAA (0.1 mg/L) and 3% maltose at 25°C in the dark for 2 days. The infected leaf disks were soaked in liquid MS medium for 5 min and then rinsed one time with liquid MS medium. The leaf disks were then blotted dry and transferred to a selection medium (MS medium supplemented with kanamycin 300 mg/L, cefotaxime 150 mg/L and 3 or 5% sucrose). Each treatment has at least 100 leaf disks with three replicates.

The selection mediums were incubated at 25°C under continuous light for 25–30 days for shoot regeneration. The transgenic shoots were transferred to rooting medium (MS medium supplemented with Kanamycin 100 mg/L and 3% sucrose).

The putative transgenic tobacco plants were confirmed by PCR with primers: PMOA36-TBS *Pme*I For, 5′-TGATAGAGTAGTTCATAT GGA-3′ and PMOA36-TBS *Pme*I Rev 5′-GCTTCC CAACCTTACCAGAG-3′. The PCR products were gel purified and sequenced at the core facility at Virginia Bioinformatics Institute.

### Bacterial genomic DNA, plasmid DNA, and plant genomic DNA isolation

Bacterial genomic DNAs were isolated using a ZR Fungal/Bacterial DNA MiniPrep™ (Zymo Research Corporation, Irvine, CA). Plasmid DNAs were isolated using an AccuPrep™ Plasmid Extraction Kit (Bioneer Corporation, Alameda, CA). Plant genomic DNAs were isolated by using the CTAB method as previously described (Zhang et al., 2013).

## Results and discussion

### Development of a sucrose-sensitive mutant *Agrobacterium* strain GV2260-*SacB/R*

**Figure 1:**
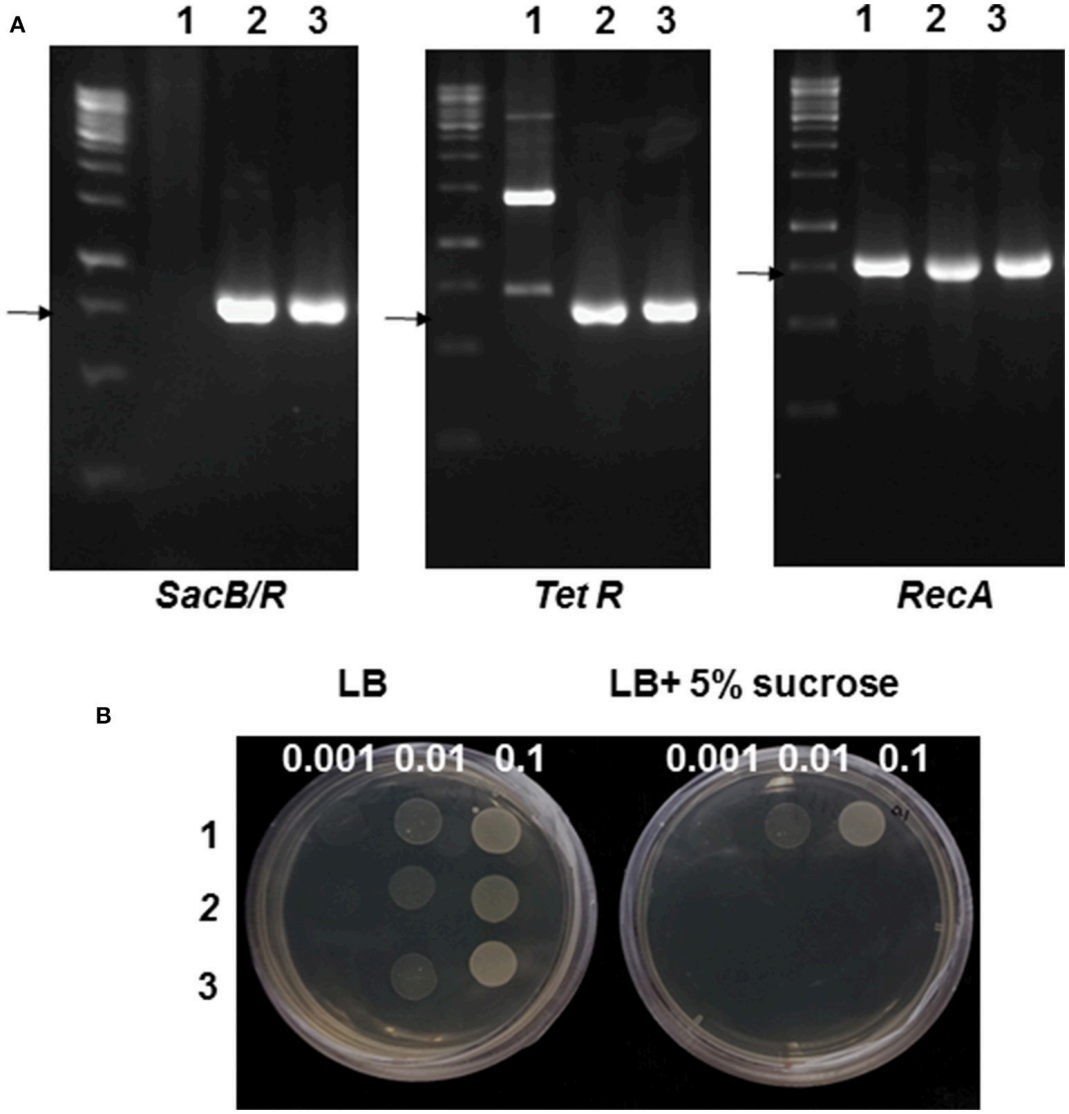
**Genotype and phenotype validation of the mutant *Agrobacterium* strain GV2260-*SacB/R***. **(A)** PCR analysis of GV2260 (1), *E. coli* carrying pLVC18L-Npt2-*SacB/R*-*recA* (2), and mutant *Agrobacterium* strain GV2260-*SacB/R* (3) with primers for detecting the tetracycline resistance gene, *SacB-SacR* and *recA* genes. The arrows highlight the specifically amplified DNA fragments. **(B)** Testing for the inhibition of GV2260 (1), *E. coli* carrying pLVC18L-Npt2-*SacB/R*-*recA* (2), and mutant *Agrobacterium* strain GV2260-*SacB/R* (3) on LB media supplemented with 5% sucrose. Three bacterial dilutions, OD600 = 0.1, 0.01, and 0.001, have been used for testing on the LB medium.

To generate a mutant *Agrobacterium* strain that is sensitive to sucrose, a suicide vector pLVC18L carrying the *SacB-SacR* gene cassette was integrated into the *recA* gene locus in the genome of *A. tumefaciens* strain GV2260 through marker-exchange mutagenesis. The mutant strain was named GV2260-*SacB/R*. The integration of the *SacB-SacR* gene cassette in GV2260-*SacB/R* was confirmed by PCR amplification. As shown in Figure 1A, the plasmid DNA of pLVC18L-*Npt*2-*SacB/R*-*recA* and the genomic DNA of GV2260-*SacB/R*, but not the wild type strain GV2260, can amplify the *SacB-SacR* gene and a tetracycline resistance gene located on the suicide vector pLVC18L-*Npt*2-*SacB/R*-*recA*. All three DNAs can amplify the *recA* gene fragment. These results suggest that GV2260-*SacB/R* is carrying the *SacB*-*SacR* gene cassette. The mutant strain GV2260-*SacB/R* is expected to carry a non-functional *recA* gene (Lovett et al., 1993; Bi and Liu, 1994). In the future, it will be interesting to test the stability of plasmids maintained in GV2260-*SacB/R*.

To test the sucrose-sensitivity of GV2260-*SacB/R*, the mutant strain along with the wild type strain GV2260, and *E. coli* strain DH5α carrying pLVC18L-Npt2-*SacB/R*-*recA* were grown on LB agar medium supplemented with or without 5% sucrose. The wild type *A. tumefaciens* strain GV2260 grew equally well on LB medium with or without 5% sucrose, which suggests that sucrose in LB agar medium has no inhibitory effect on *A. tumefaciens* GV2260 (Figure 1B). In contrast, the *E. coli* strain DH5α carrying pLVC18L-Npt2-*SacB/R*-*recA* and GV2260-*SacB/R* grew well on LB agar medium without 5% sucrose, but showed almost no growth on LB agar medium with 5% sucrose. This result suggests that *E. coli* carrying pLVC18L-Npt2-*SacB/R*-*recA* and GV2260-*SacB/R* containing the *SacB-SacR* gene cassette can be effectively inhibited by the 5% sucrose presented in the culture medium.

### Mutant *Agrobacterium* strain GV2260-*SacB/R* maintains its ability of transforming tobacco plant cells

**Figure 2:**
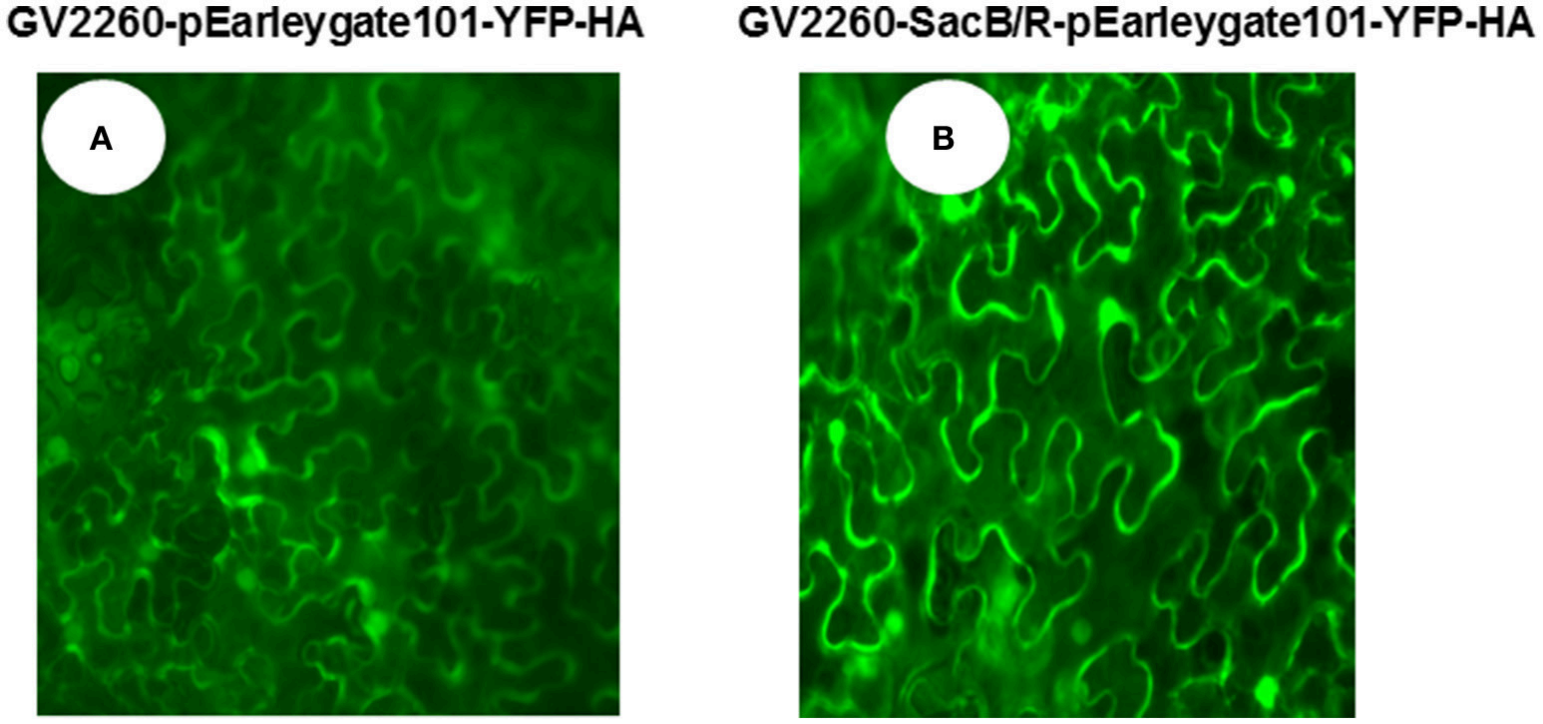
***Agrobacterium*-mediated transient expression of YFP in the leaves of *N. benthamiana.* (A)** *N. benthamiana* leaf inoculated with *Agrobacterium* strain GV2260 carrying pEarleygate101-YFP-HA, **(B)** *N. benthamiana* leaf inoculated with *Agrobacterium* strain GV2260-*SacB/R* carrying pEarleygate101-YFP-HA.

To examine if GV2260-*SacB/R* can be used for plant cell transformation, the GV2260 and GV2260-*SacB/R* strains carrying plasmid construct pEarleygate101-YFP-HA were infiltrated into the leaves of *N. benthamiana*. In this construct, the *YFP* gene was cloned behind the CaMV 35S promoter, and it can be expressed in the transformed tobacco plant cells. Strong YFP fluorescence signals were detected from leaves inoculated with either GV2260 or GV2260-*SacB/R* carrying the *YFP* gene (Figure 2), which suggests that both strains can successfully transform the *N. benthamiana* plant cells.

### Generation of stable transgenic *N. benthamiana* plants using the mutant *Agrobacterium* strain GV2260-*SacB/R*

**Figure 3:**
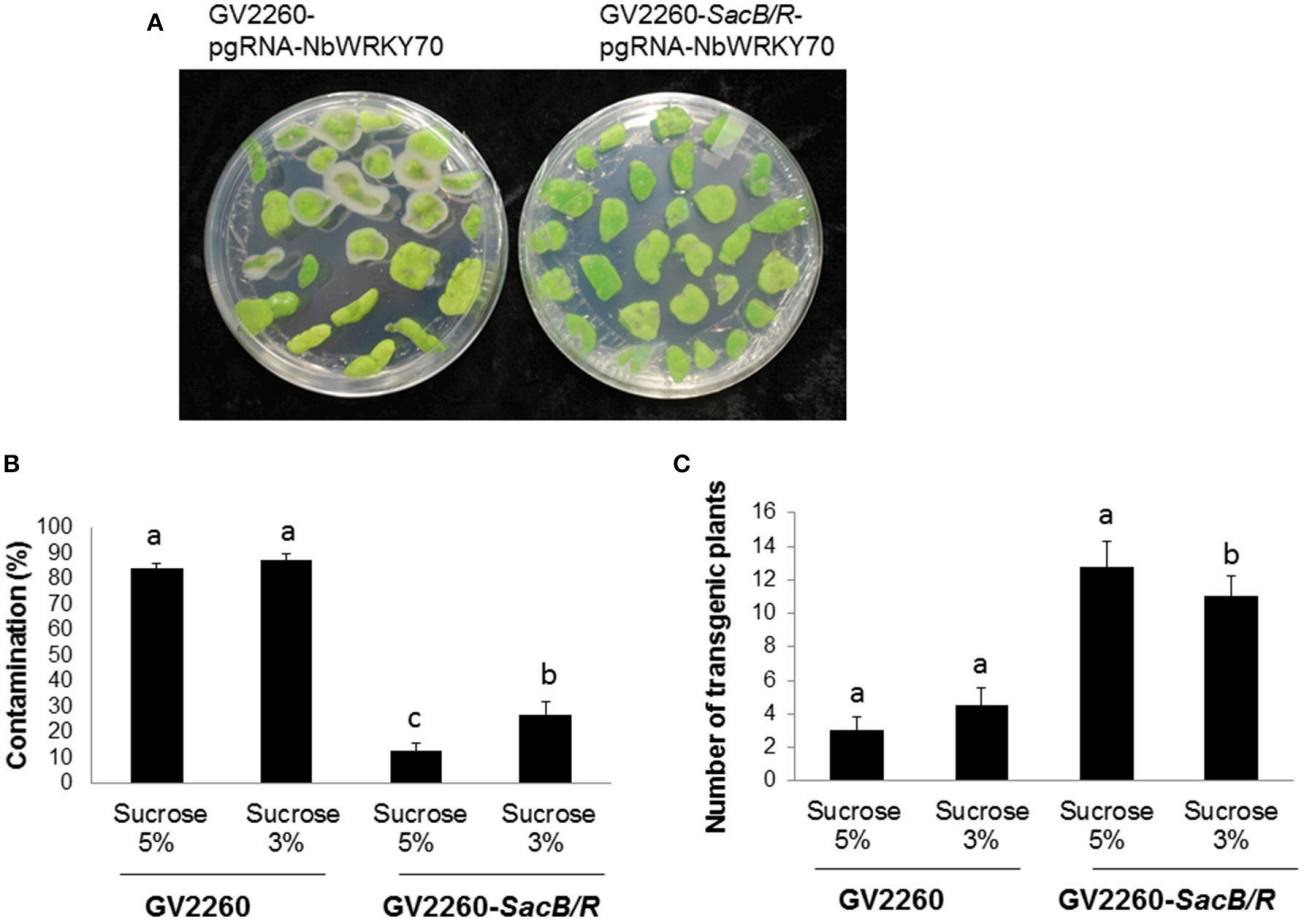
**Comparison of the contamination ratio and transformation efficiency between GV2260 and mutant *Agrobacterium* strain GV2260-*SacB/R* during *N. benthamiana* tissue culture and transformation. (A)** Leaf disks with *Agrobacterium* overgrowth. **(B)** Contamination ratio of GV2260 and mutant *Agrobacterium* strain GV2260-*SacB/R* during *N. benthamiana* transformation on tissue culture medium supplemented with 5 and 3% sucrose (Tukey HSD, *P* < 0.05). **(C)** Number of transgenic plants generated from GV2260 and GV2260-*SacB/R* (Tukey HSD, *P* < 0.05). Difference letters indicate statistical significant difference.

To test the transformation efficiency of GV2260-*SacB/R, N. benthamiana* tissue culture and transformation was conducted with *Agrobacterium* strain GV2260 or GV2260-*SacB/R* carrying plasmid pgRNA-*NbWRKY70*. The construct pgRNA-NbWRKY70 carries a synthesized *Cas9* gene driven by the *Arabidopsis* Ubiquitin 10 promoter. The expression of a guiding RNA targeting on the *N. benthamiana* WRKY70 gene was driven by the *Arabidopsis* U6 promoter. We modified the *N. benthamiana* leaf disk transformation protocol (An, 1985), where the leaf disks infected with *Agrobacterium* strains were only slightly washed, which usually can cause *Agrobacterium* overgrowth problems during the selection of transformed plant cells. The infected *N. benthamiana* leaf disks were cultured on selection medium supplemented with either 3 or 5% sucrose. The leaf-disk contamination caused by the overgrowth of *Agrobacterium* was recorded after 4 weeks of culture on the selection medium (Figure 3A). Under the test conditions, the contamination rates caused by GV2260-*SacB/R* on medium supplemented with 5 and 3% sucrose were 13.0 and 26.9% respectively, which are significant lower than the contamination rate of >80% caused by GV2260 (Figure 3B). Therefore, GV2260-*SacB/R* can be efficiently inhibited by the sucrose presented in the *N. benthamiana* tissue culture medium. However, under our testing conditions, there were still quite high numbers of contaminated leaf disks when in infections with GV2260-*SacB/R*. We speculate that the slightly rinsed leaf disks carried relatively high numbers of *Agrobacterium* cells, which may develop mutations on the *SacB-SacR* genes during the tissue culture process. It will be interesting to further test with different wash conditions, which may reduce the carry-on bacterium cells, and allow for further reduction of the contamination ratio. It will also be interesting to test if we can reduce or even eliminate antibiotic during the tissue culture process.

**Figure 4:**
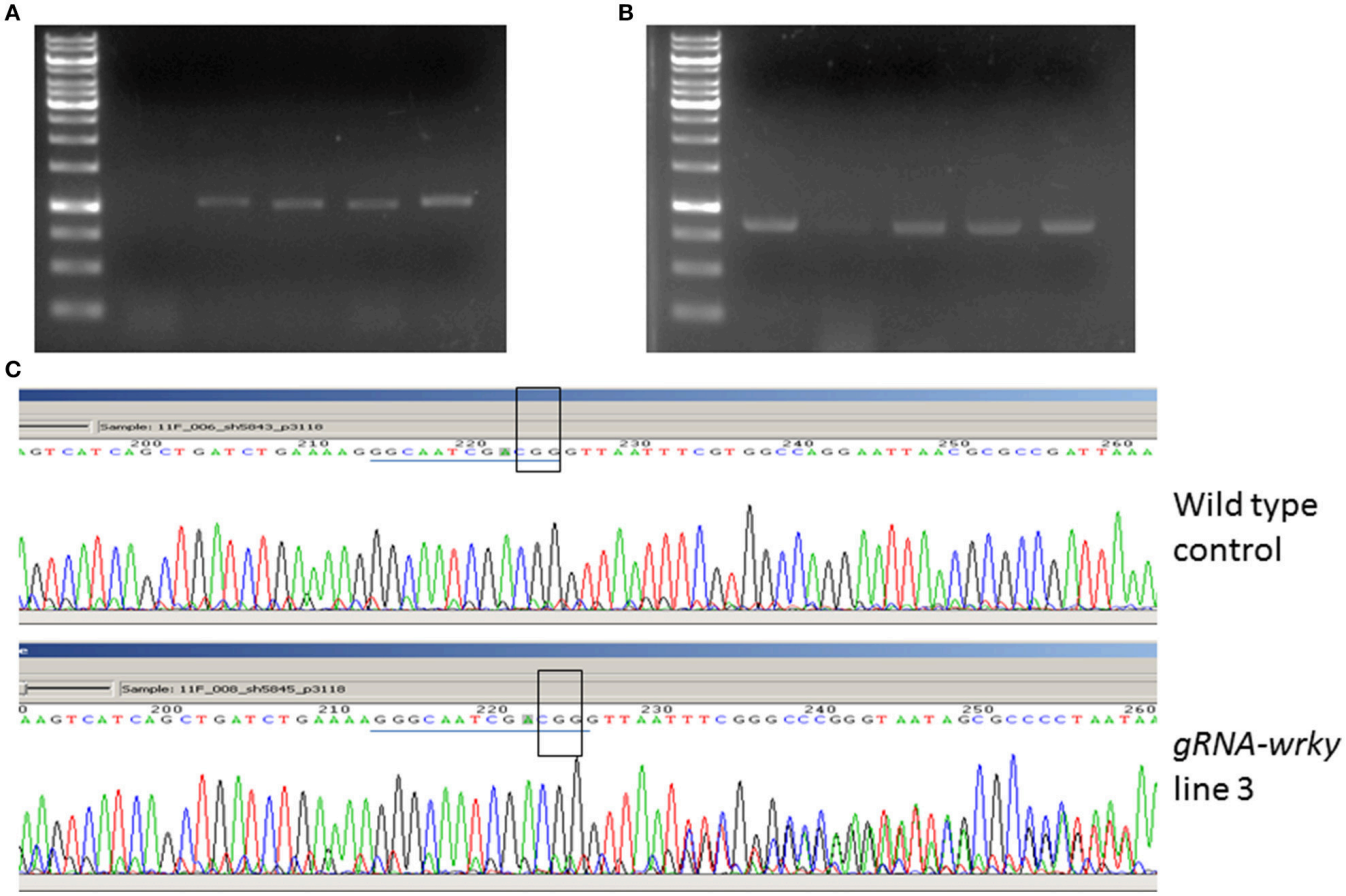
**Detection of the p*gRNA*-*NbWRKY* construct and its induced mutation. (A)** Amplification of the guiding RNA construct from transgenic and non-transgenic control. Lane 1, 1 Kb marker, lane 2, non-transgenic control, lanes 3–6, four transgenic plants. **(B)** Amplification of the Nb*WRKY* DNA fragment carrying the guiding RNA targeting site. Lane 1, 1 Kb marker, lane 2, non-transgenic control, lanes 3–6, four transgenic plants. **(C)** Sequencing of the PCR product from the wild type and gRNA-Nb*WRKY* transgenic line 3. The guiding RNA targeting sites are highlighted with a blue line, and the PAM sites are highlighted with an open box. The sequencing chromatograms showed mixed peak-signals after the PAM site in gRNA-Nb*WRKY* transgenic line 3 but not in the non-transgenic control.

After 2 months of culture selection, putative transgenic *N. benthamiana* plants were generated. The number of transgenic plants generated from GV2260-*SacB/R* was significantly higher than those generated from GV2260 (Figure 3C) because of the lower contamination rate caused by the GV2260-*SacB/R* strain. Four transgenic plants generated by GV2260-*SacB/R* were genotyped, showing the presence of the *Cas9* gene (Figure 4A). To examine if the pgRNA-*NbWRKY70* transgenic plants carry mutations in *WRKY70*, we amplified an *NbWRKY70* DNA fragment carrying the guiding RNA targeting site. All four putative transgenic lines amplified an *NbWRKY70* DNA fragment with similar size (Figure 4B). The PCR products were gel purified and sequenced. As shown in the chromatogram, the PCR product amplified from the wild type plants yields a clean sequence, while the PCR product from a transgenic plant (line 3) yields double peaks near the PAM site (CGG) (Figure 4C) (Li et al., 2011; Nekrasov et al., 2013). The double peak near the PAM site indicates the heterozygosity of template DNAs, which suggests there are *Cas9* induced mutations at the *WRKY70* gene. The PCR products were also cloned and individual clones were sequenced, which confirmed the presence of mutants (data not shown). The phenotype of transgenic plants will be further characterized in the future. Nevertheless, our result demonstrated that GV2260-*SacB/R* could successfully transform *N. benthamiana* plant cells to generate stable transgenic plants. It will be interesting to test the transformation capacity of GV2260-*SacB/R* in other plant species. In this study, we also confirmed that the CRISPR-*Cas*9 system is a powerful tool for introducing mutations on target genes in *N. benthamiana* (Nekrasov et al., 2013; Belhaj et al., 2015).

## Conclusions

We generated a mutant *Agrobacterium* strain GV2260-*SacB/R* that is sensitive to sucrose. The mutant strain can be used for plant cell transformation as demonstrated by *Agrobacterium*-mediated transient assays and stable transformation. The overgrowth of mutant strain GV2260-*SacB/R* can be inhibited by 3–5% sucrose, a common carbon source used in plant tissue mediums. Therefore, GV2260-*SacB/R* can be a valuable tool for plant transformation research.

## Author contributions

BZ conceived the project and the cloning strategy. YML and JM performed the experiments. ST, DK, YL, ZN, and XZ contributed vectors and other reagents. YML, JM, ST, DK, YL, ZN, XZ, ZL, and BZ analyzed the data and wrote the draft manuscript. YML and BZ wrote the final manuscript. All authors read and approved the final manuscript.

### Conflict of interest statement

The authors declare that the research was conducted in the absence of any commercial or financial relationships that could be construed as a potential conflict of interest.
